# Musashi-1 Enhances Glioblastoma Cell Migration and Cytoskeletal Dynamics through Translational Inhibition of Tensin3

**DOI:** 10.1038/s41598-017-09504-7

**Published:** 2017-08-18

**Authors:** Hsiao-Yun Chen, Liang-Ting Lin, Mong-Lien Wang, Benoit Laurent, Chih-Hung Hsu, Chih-Ming Pan, Wan-Ru Jiang, Pau-Yuan Chen, Hsin-I Ma, Yi-Wei Chen, Pin-I Huang, Arthur Chiou, Shih-Hwa Chiou

**Affiliations:** 1 Institute of Clinical Medicine, National Yang-Ming University, Taipei, Taiwan; 2 Stem Cell Center, Department of Medical Research, Taipei Veterans General Hospital, Taipei, Taiwan; 3 Institute of Pharmacology, National Yang-Ming University, Taipei, Taiwan; 4 Institute of Biochemistry and Molecular Biology, National Yang-Ming University, Taipei, Taiwan; 5 Institute of Biophotonics, National Yang-Ming University, Taipei, Taiwan; 6 Department of Health Technology and Informatics, The Hong Kong Polytechnic University, Kowloon, Hong Kong; 7 Boston Children Hospital and Harvard Medical School, Boston, MA USA; 8 Program in Epigenetic and Molecular Cell Biology, School of Medicine and Public Health, Zhejiang University, Hangzhou, China; 9 Center for Cell Therapy, Department of Medical Research, China Medical University Hospital, Taichung, Taiwan; 10 Institute of Biomedical Engineering, National Chiao-Tung University, Hsinchu, Taiwan; 11 Department of Neurological Surgery, Tri-Service General Hospital and National Defense Medical Center, Taipei, Taiwan; 12 Cancer Center, Taipei Veterans General Hospital, Taipei, Taiwan; 13 Genomic Research Center, Academia Sinica, Taipei, Taiwan

## Abstract

The RNA-binding protein Musashi-1 (MSI1) exerts essential roles in multiple cellular functions, such as maintenance of self-renewal and pluripotency of stem cells. MSI1 overexpression has been observed in several tumor tissues, including glioblastoma (GBM), and is considered as a well-established marker for tumor metastasis and recurrence. However, the molecular mechanisms by which MSI1 regulates cell migration are still undetermined. Here we reported that MSI1 alters cell morphology, promotes cell migration, and increases viscoelasticity of GBM cells. We also found that MSI1 directly binds to the 3′UTR of Tensin 3 (TNS3) mRNA, a negative regulator of cell migration, to inhibit its translation. Additionally, we identified that RhoA-GTP could be a potential regulator in MSI1/TNS3-mediated cell migration and morphological changes. In a xenograft animal model, high expression ratio of MSI1 to TNS3 enhanced GBM tumor migration. We also confirmed that MSI1 and TNS3 expressions are mutually exclusive in migratory tumor lesions, and GBM patients with MSI1^high^/TNS3^low^ pattern tend to have poor clinical outcome. Taken together, our findings suggested a critical role of MSI1-TNS3 axis in regulating GBM migration and highlighted that the ratio of MSI1/TNS3 could predict metastatic and survival outcome of GBM patients.

## Introduction

Glioblastoma (GBM), or grade IV astrocytoma, is the most common and fatal primary brain tumor with dismal prognosis^1, 2^. The hallmarks of aggressive GBM include diffuse migration and local invasion of tumor cells into surrounding tissues which shelter them from surgery and radiation^3^. Thus, elucidation of the molecular mechanisms underlying migration or invasion of GBM cells is critical to improve the current treatment effect.

Musashi-1 (MSI1) is a well-conserved RBP that has been previously described to modulate translation by binding to target mRNAs^4, 5^. Increasing evidence indicated that MSI1 promotes malignancy in hepatocellular carcinoma, lung cancer, cervical cancer or glioblastoma (GBM), by regulating proliferation, survival and tumorigenesis^6–10^. MSI1 overexpression modulates Notch1 and PI3 kinase/Akt signaling, leading to tumor proliferation and infiltration^11, 12^. MSI1 regulates *NUMB* translational inhibition to restrict proteasome activity and preserve the tumor initiating ability of breast and GBM cells^13^. MSI1 binds *CDKN1A* mRNA to enforce the abrogation of cell cycle checkpoints^14^. Despite the identification of potential candidates by individual approaches^6, 15, 16^, the underlying mechanisms by which MSI1 regulate invasion and metastasis of malignant tumors, especially in GBM, remain unclear and are waiting to be investigated.

Cell migration plays a critical role in many biological processes, like embryonic development, immune response or tissue repair^17–20^. And dysregulated cell migration has been implicated in inflammatory disorders, vascular diseases, cancer invasion and metastasis^21, 22^. Assembly and disassembly of filamentous actin (F-actin) regulate cell extension and retraction^23^, and are also important for migration, focal adhesion and division^24^. The regulation of cell structure is driven by many signaling proteins. The Rho family of GTPase, including RhoA and ROCK, are well-characterized effectors that control actin polymerization and microtubule stabilization^25, 26^. RhoA overexpression is found in many malignancies and is associated with invasion and poor prognosis^27^.

In this study, we demonstrated the MSI1/TNS3/RhoA-GTP axis is the major pathway that regulates migration of GBM cells. Overexpression of MSI1 in GBM cells promotes their mobility and migration, in combination with changes in cell morphology, viscoelasticity and flexibility. By RIP-seq, we identified Tensin 3 (TNS3) as a MSI1 target mRNA. Our results indicated that MSI1/TNS3 pathway controls cell migration and morphological changes through RhoA-GTP activation. *In vivo* xenograft model confirmed that the ratio of MSI1/TNS3 expression is important for GBM tumor migration. Furthermore, we found that MSI1 and TNS3 expressions are mutually exclusive in migratory tumor lesions and MSI1^high^TNS3^low^ tumor pattern correlates with poor prognosis for GBM patients These data suggested that MSI1/TNS3 expression ratio could serve as a possible marker to predict survival outcome of GBM patients.

## Results and Discussion

### MSI1 expression increases migration and aspect ratio of GBM cells

**Figure 1:**
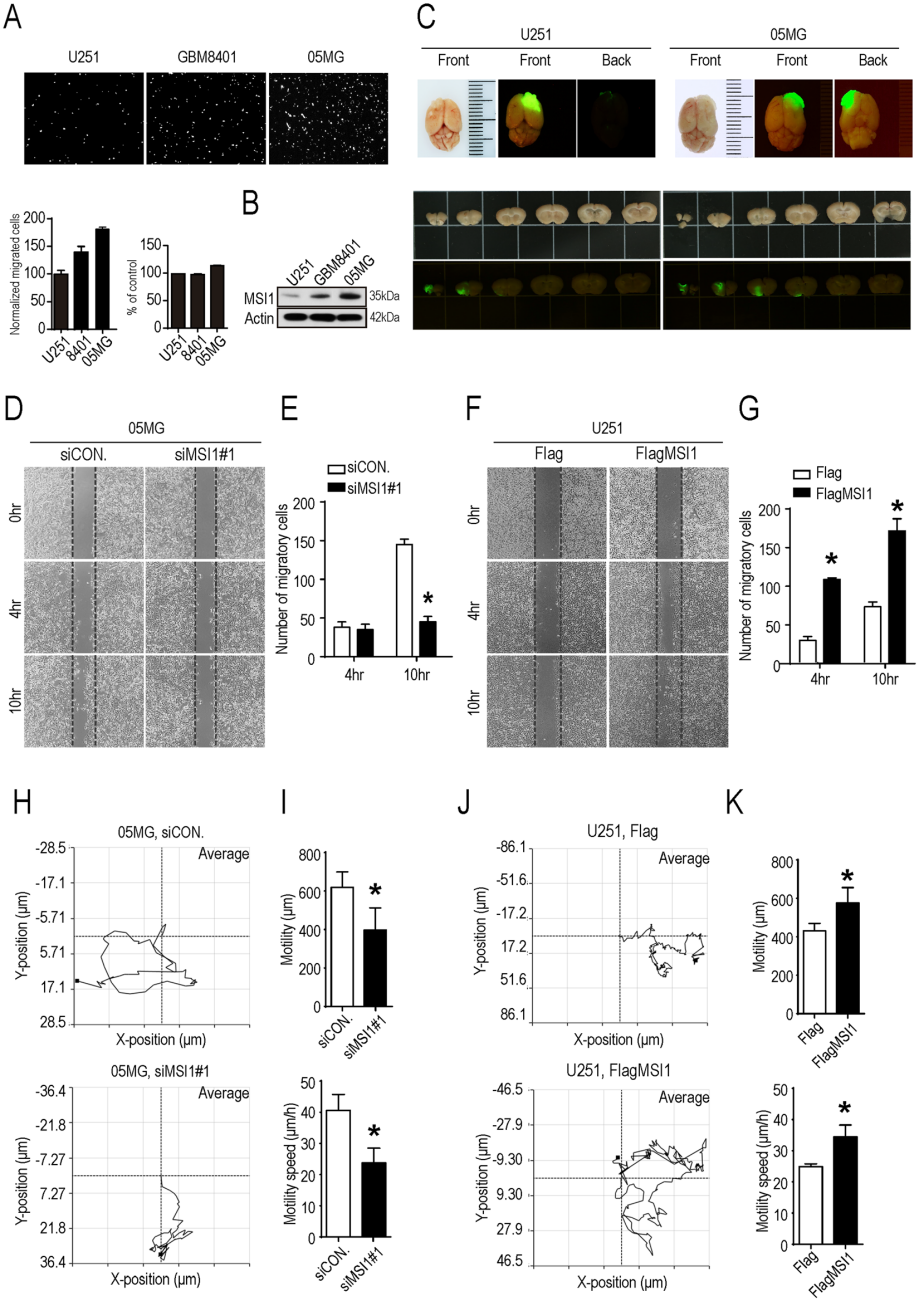
MSI1 promoted GBM cells migration. (**A**) U251, GBM8401, and 05MG GBM cell lines were subjected to a 24-hour Transwell migration assay. Cells were plated in the upper chamber, after 24 hours plating, migrating cells that moved to the underside of the filters were fixed and stained with PI (Propidium iodide). Relative cell migration was determined by the number of the migrated cells normalized to the total number of the cell, and the value from U251 cell was arbitrarily set at 100%; the percentage of cell numbers were indicated by alamar blue assay shown in bar chart. (**B**) Western blot analysis of MSI1 expression in U251, GBM8401 and 05MG cell lines, of which the uncropped blots were demonstrated in Supplementary Figure 5. (**C**) SCID mice were orthotopically transplanted with 1.5 × 10^5^ GFP-labeled U251 or 1.5 × 10^5^ GFP-labeled 05MG cells. Representative tumor photographs of GFP images were taken 63 days after transplantation. (**D**,**E**) 05MG cell transiently transfected with siRNA against MSI1 (siMSI1) or scrambled siRNA control (siCON.), and the number of migrated cells at 4 and 10 hours were calculated. (**F**,**G**) U251 cells were transiently transfected with Flag-tagged MSI1 (FlagMSI1) or Flag control (Flag), and the number of migrated cells at 4 and 10 hours were presented as the bar chart. MSI1-depleted 05MG (**H**,**I**) and MSI1-overexpressed U251 cells (**J**,**K**) were seeded in 3.5-cm plates prior to the image acquisition using time lapse microscopy for single-cell tracking (1 frame/5 min for 24 hours). The mean tracks of 15 cells were shown in the left panel. The mean motility and speed of cells was determined by the manual tracking plugin of Image J in three independent analyses and shown as bar chart in the right. Each column and bar showed the mean ± SEM. The experiments were repeated at least three times. The statistical significance was assessed by one-way ANOVA, *P < 0.05 (relative to the control group).

High level of MSI1 expression has been associated with GBM malignancy and poor survival of patients^28, 29^. However, the link between MSI1 and GBM cell migration has not been clearly defined. To investigate this point, we firstly carried out a transwell assay to evaluate the migration ability of three GBM cell lines: U251, GBM8401, and 05MG. Our results demonstrated that 05MG cells exhibited the strongest migration ability while U251 cells showed a limited capability of migration (Fig. 1A). And this indicated the percentage of migrating cells was positively correlated with the level of MSI1 expression (Fig. 1B). For *in vivo* investigation, mice were orthotopically transplanted with GFP-labeled U251 or GFP-labeled 05MG cells, expressing lower and higher level of MSI1 proteins, respectively. The post-mortem study of the brains clearly showed that, contrary to U251 cells, GFP-labeled 05MG cells were present much deeper into the basal skull (Fig. 1C), suggesting that high expression of MSI1 could contribute to tumor invasiveness and cell migration.

To confirm the effects of MSI1 on cell migration, we then knocked down MSI1 in 05MG cells using siRNA (siMSI1#1 and siMSI1#2). Wound-healing and transwell assays both showed that cell migration was significantly impeded in absence of MSI1 (Fig. 1D,E and Fig. S1A,B). On the other hand, overexpression of Flag-tagged MSI1 (FlagMSI1) in U251 cells increased cell migration (Fig. 1F,G and Fig. S1C,D). To precisely measure the movement of an individual cell, a single-cell migration trace recording was conducted for 24 hours. We showed that inhibition of MSI1 reduced the mobility and speed of 05MG cells (Fig. 1H,I) while its overexpression in U251 cells increased mobility and speed of the cells (Fig. 1J,K). Altogether, these results indicated that MSI1 expression promotes GBM cell mobility and migration both *in vitro* and *in vivo*.

**Figure 2:**
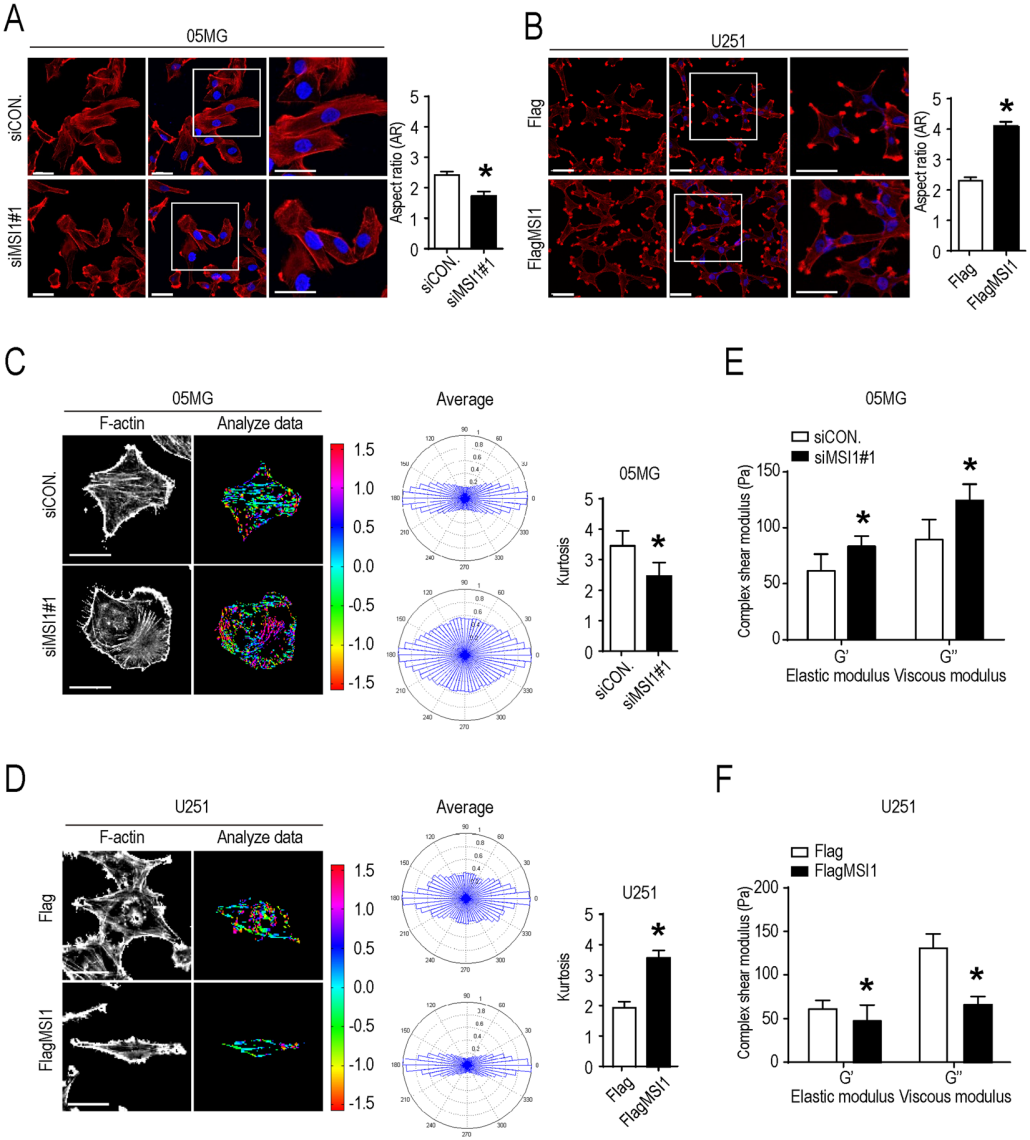
MSI1 increased the aspect ratio and decreased viscoelasticity of GBM cells. (**A**)(**B**) MSI1-depleted 05MG cells and MSI1-overexpressed U251 cells were immune-stained for F-actin (Red) and DAPI (Blue) and observed by fluorescent microscopy. The ratio of mean aspect (defined as the ratio of the length of major and minor axes, AR) were shown in the right (n = 100). (**C**)(**D**) Fluorescent color maps of F-actin networks distribution in MSI1-depleted 05MG cells and MSI1-overexpressed U251 cells. The formation and the angular distribution of F-actin orientation in MSI1-depleted 05MG and MSI1-overexpressed U251 cells were analyzed by assessing and quantifying the immunofluorescence intensities using MatLab software. The mean of Kurtosis from 45 cells was shown in the right. (**E**) (**F**) The intracellular elastic modulus (**G**’) and viscous modulus (**G**”, at 10 Hz) of MSI1-depleted 05MG and MSI1-overexpressed U251 cells were shown as mean ± SEM (n = 100 beads/ 35cells). Each bar chart is presented as mean ± SEM. The experiments were repeated at least three times. The statistical significance was assessed by one-way ANOVA, *P < 0.05 (relative to the control group).

Cell migration is physically mediated by actin cytoskeleton reorganization and initiated by the protrusion of the cell membrane^30^. We stained F-actin with immunofluorescence in control and MSI1-knockdown (KD) 05MG cells and then quantified the intensity to determine the angular distribution of F-actin. As shown in Fig. 2A–D, actin filaments were well-aligned with the cell major axis in control cells whereas the filaments were reoriented to occupy a more uniform and rounded shape distribution in MSI1-knockdown cells. Quantification of the morphological changes revealed that aspect ratio and Kurtosis coefficient were significantly decreased in the MSI1 KD-05MG cells (Fig. 2A,B). And the opposite results were observed in control and MSI1-overexpressing U251 cells (Fig. 2C,D). Moreover, we measured the elasticity (G’) and viscosity (G”) parameters using video particle tracking microrheology (VPTM). In Fig. 2E,F showed that MSI1-KD 05MG cells increased the elasticity and viscosity but this phenomenon was not occurred in MSI1-overexpressing U251 cells, suggesting that MSI1 promotes viscoelasticity, and flexibility of GBM cells. Our data concluded that high expression of MSI1 contributes to cell mobility speed and migration, in combination with important changes in morphology, viscoelasticity and flexibility of GBM cells.

### MSI1 binds mRNAs associated with cell migration and adhesion pathways

**Figure 3:**
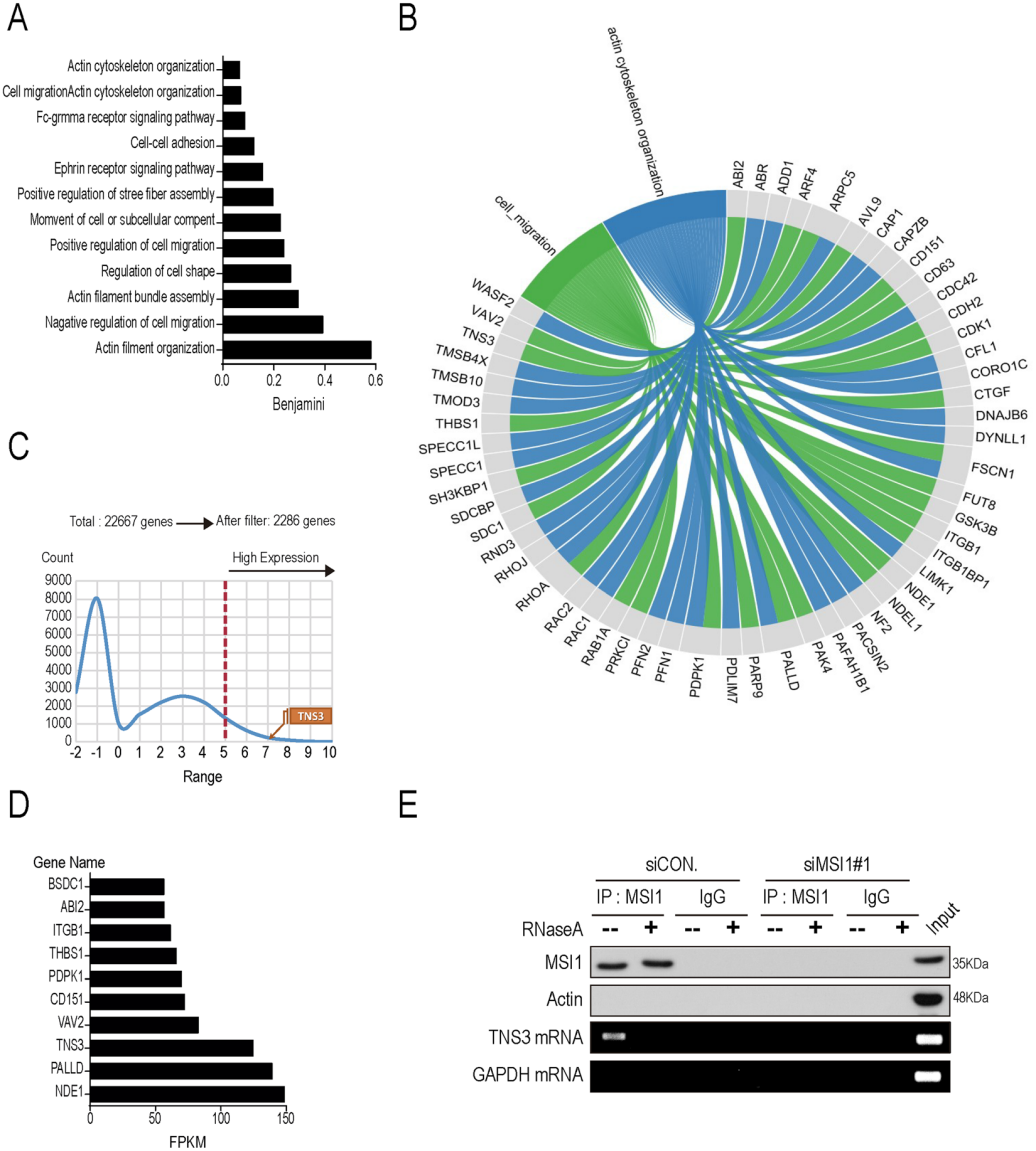
Gene ontology (GO) and pathway analyses for MSI1 targets distribution in actin rearrangement. Ribonucleoprotein immunoprecipitation sequencing (RIP-seq) of MSI1-associated RNAs were performed in 05MG cells. (**A**) Gene ontology (GO term) enrichment analysis was conducted by DAVID software per the biological processed. (**B**) MSI1-immunoprecipitated mRNAs are enriched for protein-protein interaction network, of which the top-ranked genes from the GO terms were listed. (**C**) Non-significant genes were cut off, narrowing down the original 22667 genes into 2286 target genes. TNS3 was found in the highly expressed pool of genes. (**D**) The top 10 significantly changed genes were analyzed by REVIGO (http://revigo.irb.hr/) and listed in the chart. The numbers on each graph showed the corresponding GO term of cell migration and its read counts by FPKM (fragments per kilobase of exon per million fragments mapped). (**E**) Endogenous MSI1 was precipitated from 05MG cells treated without or with RNaseA (10 mg/ml). The precipitated complexes were subjected to RT-PCR to assess TNS3 mRNA levels and by Western blotting to confirm the MSI1 precipitation, of which the uncropped blots were demonstrated in Supplementary Figure 5.

To decipher the molecular mechanisms at mRNA level, we conducted RNA immunoprecipitation experiments followed by sequencing (RIP-seq) on parental GBM cells. Bioinformatics analyses identified a total number of 2286 target mRNAs that were significantly enriched in the MSI1-immunoprecipitated RNA pool compared to the control. Gene Ontology (GO) analyses revealed that these transcripts were functionally enriched in actin cytoskeleton organization and cell migration (Fig. 3A,B and Fig. S2A), suggesting that MSI1 could regulate the migratory machinery in GBM cells. By using previous RNA-seq and microarray data^29^, we discovered that genes involved in the cell migration pathway were highly expressed (Fig. 3C,D and Fig. S2B). Three genes, including TNS3, PALLD, and NDE1, were identified as putative target mRNAs with the highest expression in GBM cells (Fig. 3D). After cross-checking with the result of previous RIP-seq and microarray, we chose Tensin 3 (TNS3) as the target mRNA in our model TNS3 has been found to negatively regulate cell migration, cytoskeletal dynamics and related processes such as cell shape, process formation, and motility^31^. Its overexpression inhibits cell migration while knockdown of it significantly increases migration of cancer cells^30, 32^. Results from the RIP-seq experiment showed the association between MSI1 protein and *TNS3* mRNA in 05MG control cells but not in MSI1-KD cells (Fig. 3E).

### MSI1 represses TNS3 translation through its binding to the 3′UTR

**Figure 4:**
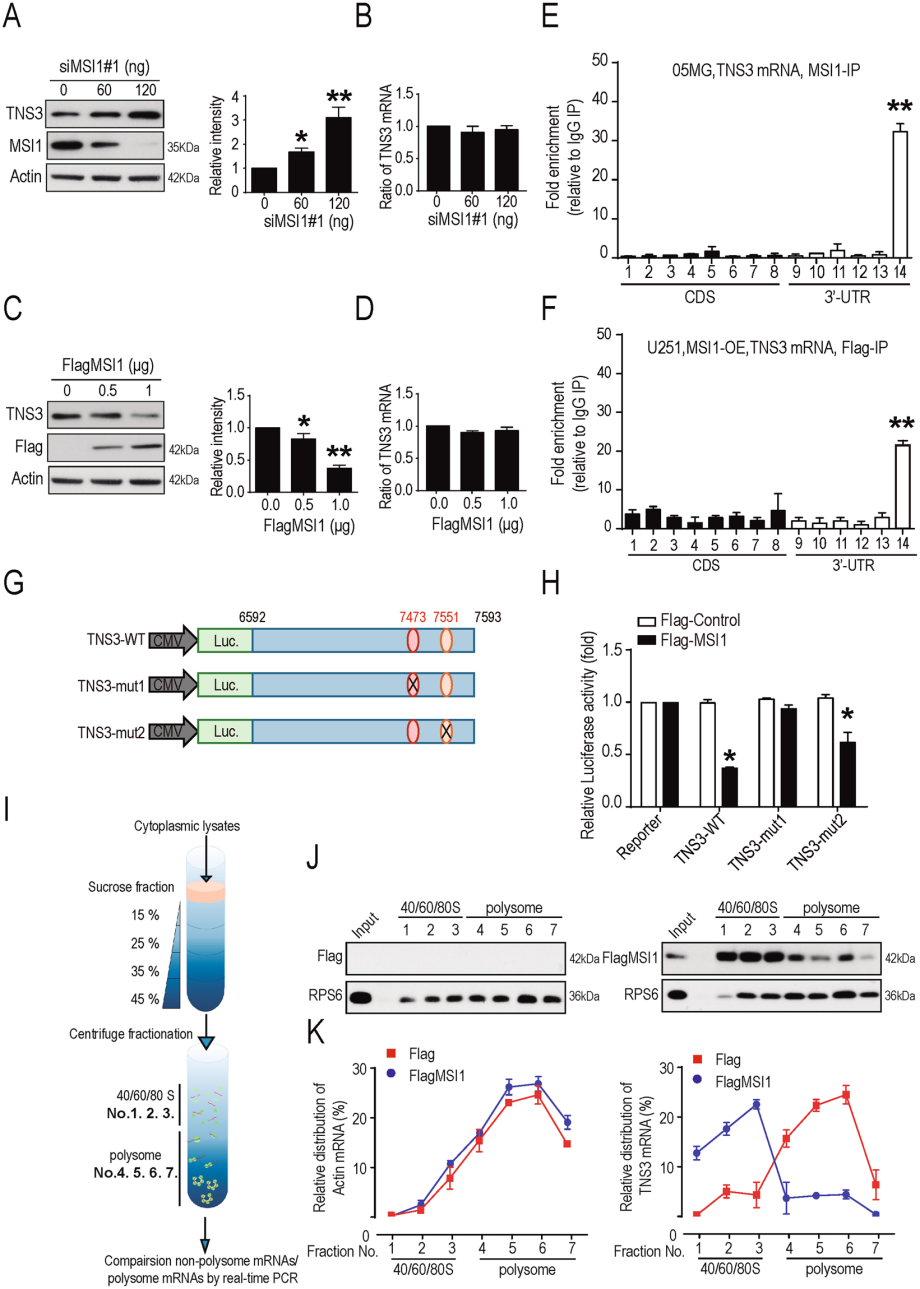
MSI1 repressed TNS3 translation through binding to its 3′UTR. GBM 05MG cells were transiently transfected with 0, 60 and 120 ng of siMSI1. TNS3 and MSI1 expression levels were analyzed 48 hours after transfection by (**A**) Western blotting and (**B**) Real-time PCR. U251 cells were transient transfected with 0, 0.5 and 1 μg Flag-MSI1. TNS3 and MSI1 expression levels were analyzed 48 hours after transfection by Western blotting (left), and the results were quantified as bar chart (right) and Real-time PCR (**D**). (**E**,**F**) RNA-ChIP assay was performed in endogenous MSI1 in 05MG cells and exogenous Flag-MSI1 in MSI1-overexpressed U251 cells. The bar chart indicates the gene expression fold change after normalization to IgG-precipitated controls. (**G**) Schematic representation of the reporter constructs containing the firefly luciferase fused to wild-type or mutated TNS3-3′UTR. (**H**) Luciferase reporter assays were conducted in U251 cells with or without MSI1 expression. The bioluminescent signal of each TNS3-3′UTR reporter were shown as relative fold change to their respective luciferase positive control. (**I**) Schematic illustration showing the sucrose gradient centrifugation. The gradient used in this study was 15% at the top and 45% at the bottom. Whole-cell lysates are prepared and layered carefully on top of the gradient. After centrifugation, the top 3 fractions (No. 1 to 3) of the gradient were dissembled ribosomal subunits (40/60/80 S, non-translation fraction,) and the bottom 4 fractions (No. 4 to 7) were the assembled ribosomes (polysome, translation fraction). (**J**,**K**) Flag-control and MSI1-overexpressed U251 cells were subjected to sucrose gradient centrifugation. Samples from each fraction were analyzed by Western bolt. Relative levels of TNS3 and actin mRNAs in each ribosome fraction were quantified and plotted as a percentage relative to the total input. The uncropped blots were demonstrated in supplementary figure 5. Data are from three independent polysomic-profiling experiments. Error bars represent mean ± SEM; n = 3. *p < 0.05; **p < 0.01 (relative to the control group).

As MSI1 promotes cell migration and TNS3 is a negative regulator of cell migration^30, 32^, we hypothesized that MSI1 negatively regulates TNS3 to support cancer cell migration and invasion through the binding to TNS3 mRNA to regulate its translation. To test our hypothesis, we knocked down MSI1 (siMSI1 #1 and siMSI1 #2) in 05MG cell line and showed that knockdown of MSI1 promoted expression of the TNS3 protein (Fig. 4A and Fig. S3A). However, knockdown of MSI1 had no effect on *TNS3* mRNA level (Fig. 4B), suggesting that MSI1 might inhibit TNS3 mRNA translation rather than degradation. In contrast, overexpression of MSI1 in U251 cell line showed a significant decrease of TNS3 protein level (Fig. 4C) while the mRNA level remained stable (Fig. 4D). Altogether, these results support our hypothesis that MSI1 might regulate TNS3 in a translational control.

To study the mechanisms of MSI1-TNS3 regulation, we first dissected the region of TNS3 mRNA that was bound by MSI1. RNA chromatin immunoprecipitation (RNA-ChIP) analysis followed by real-time PCR were performed in 05MG and MSI1-overexpressing U251 cell lines and showed that MSI1 targeted the 3′UTR region of TNS3 mRNA (Fig. 4E,F and Fig. S3B). MSI1 has been reported to bind to the 3′UTR of its target RNAs at a consensus sequence (G/A)U_1–3_AGU^33^. Bioinformatics analyses revealed two MSI1 typical binding sites in this target region of TNS3 mRNA in position 7473 to 7478 and 7551 to 7555 (Fig. S3C). To map the potential MSI1 binding site on 3′UTR region of TNS3 mRNA, we constructed luciferase reporter plasmids driven by 3′UTR of TNS3 with wild-type (WT) or mutant binding site (mut1 and mut2) and compared their transcriptional activities in U251 cells (Fig. 4G). We found that the luciferase activity was significantly decreased in cells co-expressing FlagMSI1 and TNS3-WT, compared with the control Flag (Fig. 4H). Mutation of the first potential MSI1 binding site (TNS3-mut1) abolished this effect while the mutation of the second binding site (TNS3-mut2) had a moderate effect on the luciferase activity (Fig. 4H), suggesting that MSI1 regulates TNS3 translation through its binding at position 7473 to 7478 in the 3′UTR region.

To confirm that MSI1 regulates the translation of TNS3 mRNA, we fractionated with a sucrose gradient the cytoplasmic lysates of U251 cells expressing Flag control and Flag MSI1, and analyzed the distribution of TNS3 mRNA in the different fractions by Real-time PCR (Fig. 4I). TNS3 mRNA was predominantly presented in the heavy fractions which contain polysomes and represent active translation. When MSI1 was overexpressed, TNS3 mRNA shifted to light fractions that contain separated subunits of the ribosome and represent inactive translation (Fig. 4J,K), whereas the internal control, actin mRNA, did not change. Additionally, using cycloheximide (CHX) treatment, the half-life of TNS3 proteins was not changed, confirming that MSI1 only affects TNS3 translation and not its protein stability (Fig. S3D,E). Taken all together, our findings showed that MSI1 binds the 3′UTR region of TNS3 mRNA to inhibit its translation activity.

### MSI1/TNS3 pathway regulates cell migration through RhoA-GTP activation

**Figure 5:**
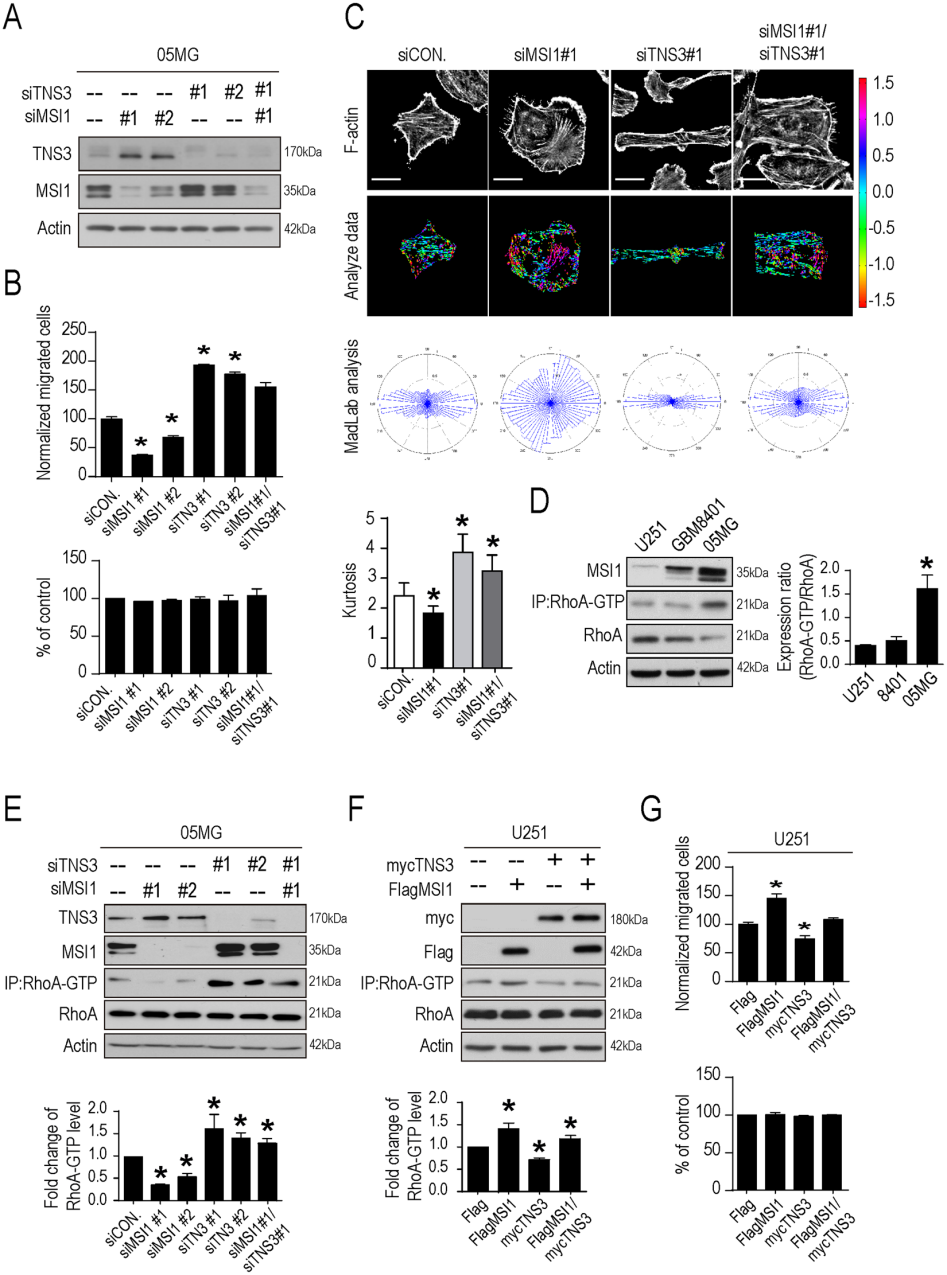
MSI1/TNS3-regulated GBM cell migration and actin remodeling is potentially governed by RhoA-GTP activation. (**A**) MSI1 and TNS3 protein levels were assessed in 05MG cells transiently transfected with siMSI1 (clone #1 and #2) and/or siTNS3 (clone #1 and #2) using Western blotting. (**B**) MSI1- and TNS3-depleted 05MG cells were subjected to TransWell migration assay. The number of migratory cells were counted and presented as percentages relative to the controls. The percentage of cell numbers were indicated by alamar blue assay shown in bar chart. (**C**) MSI1-mediated actin remodeling with TNS3 inhibition were shown with the immune-stained F-actin distribution in MSI1- and TNS3-depleted 05MG cells (left); the formation and the angular distribution of F-actin orientation were analyzed by assessing and quantifying the immunofluorescence intensities using Matlab software. Mean of Kurtosis was shown on the right (n = 45). (**D**) GTP-bound RhoA was pulled down (IP) with Rhotekin-RBD agarose beads from cell lysates and blotted with anti-RhoA antibodies. The intensities of RhoA-GTP were normalized to that of total RhoA and presented as relative fold change in the graph. (**E**) MSI1- and TNS3-depleted 05MG cells were subjected to Western blot for MSI1, TNS3 and RhoA protein levels. The intensity of RhoA-GTP blot at each group be calculating and presented as a relative ratio in comparison to the control group (siCON.). (**F**) TNS3-overexpressed (mycTNS3) and MSI1-overexpressed (FlagMSI1) U251 cells were subjected to Western blot for Flag-tag, myc-tag and RhoA protein levels. The intensity of RhoA-GTP blot was calculated and presented as a relative ratio compared to control (Flag). (**G**) Transwell migration assay was performed in MSI1-overexpressed and TNS3-overexpressed U251 cells. The number of migratory cells were counted and presented as percentage relative to the control group (Flag), while the cell numbers were indicated by alamur blue assay shown in bar chart. The uncropped blots were demonstrated in Supplementary Figure 4A. Each result is shown as the mean ± SEM. The experiments were repeated at least three times. *p < 0.05.

To determine the importance of MSI1/TNS3 co-regulation in the migration of GBM cells, we knocked down MSI1 (siMSI1#1 and siMSI1#2), TNS3 (siTNS3#1 and siTNS3#2) or both (siMSI1#1/siTNS3#2) in 05MG cells (Fig. 5A), and analyzed the cell migration capability. Transwell and Wound healing migration experiments showed that, compared with the control cells, MSI1-KD cells resulted in suppressed migration as previously described in Fig. 1D,E while TNS3-KD resulted in the increased migratory ability of GBM cells as already suggested in another cellular model^30^ (Fig. 5B and Fig. S4A–C). Consistent results were obtained for the motility (Fig. S4D,E), the morphological changes (Fig. 5C and Fig. S4F) and the viscoelasticity (Fig. S4G). Importantly, MSI1/TNS3 KD cells exhibited similar migratory ability, motility speed and morphology than the control cells (Fig. 5B,C and Fig. S4A–G), suggesting that inhibition of MSI1 was able to partially rescue the effects TNS3 inhibition. This result was quite unexpected as TNS3 is a downstream target of MSI1, leading us to hypothesize that MSI1 might directly influence a downstream effector of TNS3.

One known effector of TNS3 is the small GTPase RhoA. TNS3 inhibits the activation of RhoA, which regulates actin arrangement and promotes cell migration^26^. We analyzed the level of active RhoA (RhoA-GTP) in three GBM cell lines and showed that the level of RhoA-GTP correlates with the level of MSI1 (Fig. 5D). Western blot data showed that knockdown of MSI1 dramatically decreased the level of RhoA-GTP while knockdown of TNS3 increased it (Fig. 5E), suggesting that level of RhoA-GTP correlates with migration ability and cell morphology (Fig. 5B,C). We also concluded that MSI1 was important for RhoA activation as suggested by the strong decrease of RhoA-GTP level in MSI1-KD cells but not strictly necessary as MSI1/TNS3-KD cells exhibited a high level of RhoA-GTP, similar to that of TNS3-KD cells (Fig. 5E). We then compare these results in the context of the MSI1/TNS3/RhoA-GTP pathway by introducing TNS3 or MSI1 over-expression in U251 cells. As expected, overexpression of MSI1 significantly increased RhoA-GTP level whereas overexpressed TNS3 decreased in comparing to the control group. The concurrent over-expression of both MSI1 and TNS3 withdrew the RhoA activation (Fig. 5F) whereas overexpressing TNS3 consistently decrease their ability to migrate (Fig. 5G), suggesting that the level of RhoA-GTP correlates to the migratory ability. Collectively, our findings implied that MSI1/TNS3 axis regulates cell migration and morphology, at least partially, through RhoA-GTP activation.

### MSI1/TNS3 pathway regulates GBM tumor progression *in vivo*

**Figure 6:**
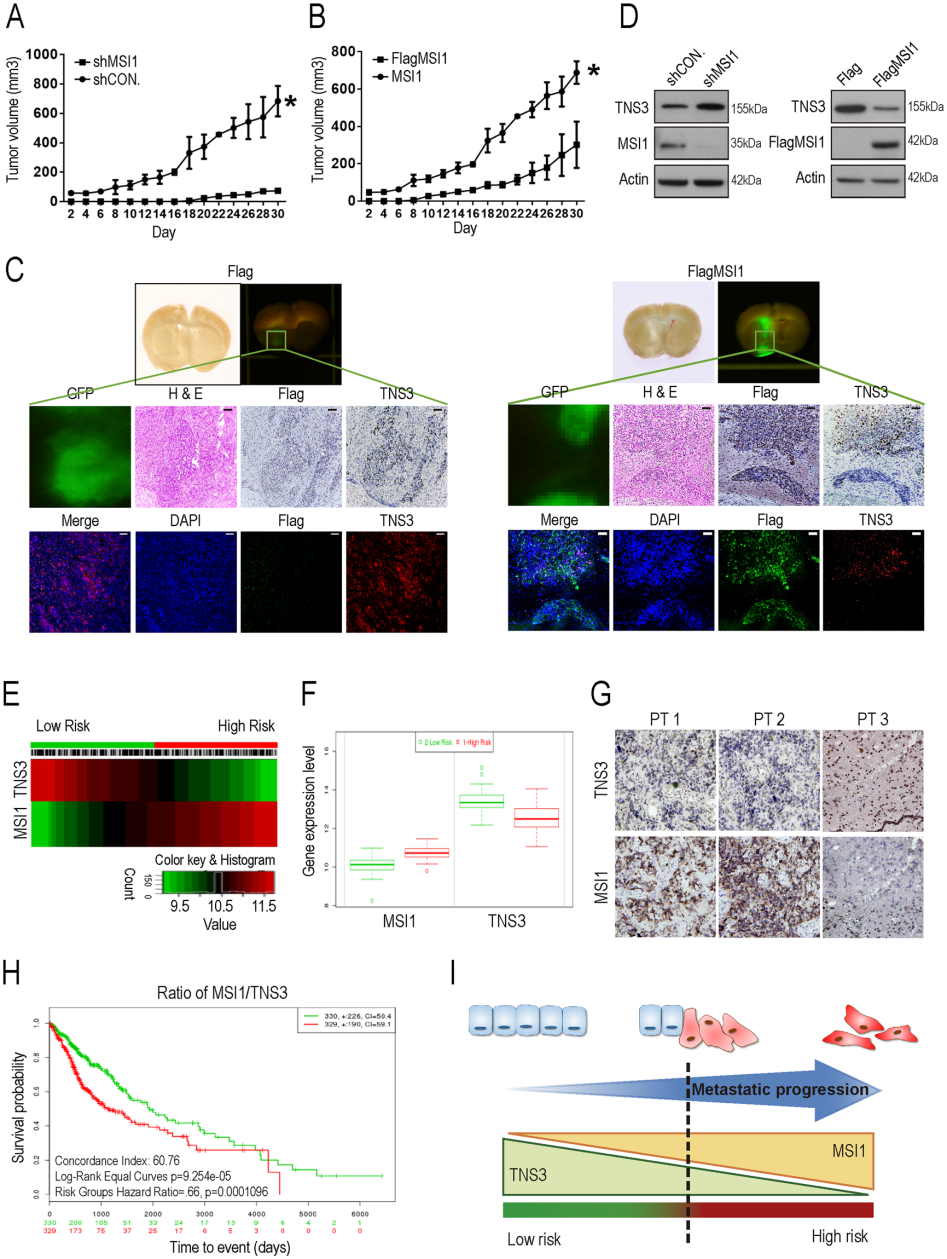
MSI1/TNS3 signaling promoted *in vivo* tumor migration and correlated with poor patient survival. (**A**,**B**) *In vivo* GBM growth rate was assessed by subcutaneous tumor implantation in immunocompromised mice with 5 × 10^5^ MSI1-depleted 05MG cells or 5 × 10^5^ MSI1-overexpressed U251 cells, each facilitated with their respective controls. Tumor volume was measured by caliper. Data were presented as mean ± SEM (n = 12). *p < 0.05 (relative to the control group). (**C**) SCID mice were orthotopically implanted with 1.5 × 10^5^ GFP-labeled U251-Flag-control or 1.5 × 10^5^ GFP-labeled U251-MSI1-ovexpressed cells. Representative photographs of fluorescent images were taken 63 days after inoculation. The GFP-labeled GBM tumors can be observed and the tumor sections were subjected to H&E, IHC, and IF staining to assess tumor malignancy, spreading, as well as the expression of MSI1 and TNS3. (**D**) Tumor tissue was harvested and homogenized for western blotting to assess MSI1 and TNS3 protein levels *ex vivo*. The uncropped blots were demonstrated in Supplementary Figure 5. Correlation of survival rates and MSI1/TNS3 gene expression in GBM patients. (**E**) Heat map of MSI1 or TNS3 mRNA expression of GBM patient samples along with the risk factor. The high and low risk groups were split of the same size depending on the Prognostic Index estimated by beta coefficients multiplied by gene expression values. (**F**) Box plots generated by SurvExpress showed the expression levels of MSI1 or TNS3 and the P-value resulting from a T-test of the difference, low-risk was in green and high-risk was in red, respectively. (**G**) Immunohistochemical staining to determine the expression level of MSI1- or TNS3-positive cells (brown color) on paraffin-embedded specimens from GBM patients (magnification × 400). (**H**) Kaplan-Meier survival curves were constructed by using SurvExpress program to analyze samples of GBM from TCGA (The Cancer Genome Atlas). The ratio of MSI1/TNS3 for Low- and High-expression groups were shown in green and red, respectively. The insets showed the number of individuals, the number censored, and the CI (confidence interval) of each risk group. (**I**) In the presented model, MSI1^high^TNS3^low^ could be used for the prediction of tumor metastatic potential and the survival outcome of patients.

Our results highlighted that the balance between MSI1/TNS3 expressions appears to be critical to regulate cell migration. To confirm our findings, we next investigated the regulatory effects of the MSI1/TNS3 pathway on GBM tumor progression. Control and MSI1 KD 05MG cells, or Flag-control and Flag-MSI1-overexpressing U251 cells were subcutaneously transplanted into immunocompromised mice and the size of the tumors was monitored during 30 days. We showed that, compared with control cells, inhibition of MSI1 in 05MG cells decreased the rate of tumor growth (Fig. 6A). On the contrary, overexpression of MSI1 in U251 cells strongly increased tumor volume (Fig. 6B). As xenograft tumors were GFP-labeled, tumor sections were then prepared and analyzed under a fluorescent microscope. We found that tumors derived from MSI1-overexpressing U251 cells were not only bigger compared to the control ones, but penetrated into the basal skull as well (Fig. 6C). Tumor tissues were also harvested and analyzed by western blot to assess MSI1 and TNS3 protein levels. We showed that TNS3 protein level inversely correlated with MSI1 expression (Fig. 6D). Immunostaining of tumor sections with Flag and TNS3 antibodies confirmed that Flag-MSI1 overexpression led to a decrease of TNS3 protein level in U251-derived tumors, compared with control (Fig. 6C). Moreover, we noticed that TNS3 staining was only observed in the local not the metastatic part of GBM tumors, despite the observation that Flag-MSI1 was expressed in both (Fig. 6C), suggesting that the expression ratio between MSI1 and TNS3 was important for the metastatic progression of the tumor.

We next analyzed the expression level of MSI1 and TNS3 in GBM patient database and showed that patients with tumors expressing a high level of MSI1 and low level of TNS3 exhibited high risk of metastasis (Fig. 6E,F). Immunostaining experiments for MSI1 and TNS3 were also performed on GBM patient samples and confirmed the inverse correlation between MSI1 and TNS3 expression (Fig. 6G). We next explored the correlation between MSI1/TNS3 expression ratio and the survival rate of patients and found that patients with a high MSI1/TNS3 ratio (high expression of MSI1 and low level of TNS3) exhibited low survival rate (Fig. 6H). Collectively, these results revealed an inverse correlation between MSI1 and TNS3 expression levels in tumors of GBM patients, and highlighted that high MSI1/TNS3 ratio could predict the metastatic potential of the tumor and therefore the survival outcome of patients.

MSI1 is a well-established marker for metastatic cancer, as a high level of MSI1 expression has been associated with GBM malignancy and poor survival of patients. The main finding of this study was that MSI1 expression promotes mobility speed and migration of GBM cells, in combination with important changes in cell morphology, viscoelasticity and flexibility. The expression level of MSI1 positively correlated with the percentage of migrating cells (Fig. 1A,B). More importantly, we showed that mice orthotopically transplanted with GBM cells expressing a high level of MSI1 exhibit much deeper invasion of GBM cells into the basal skull compared to mice transplanted with cells expressing a low level of MSI1 (Fig. 1C), confirming that high expression of MSI1 contributes to tumor invasiveness and cell migration.

At the mechanistic level, we demonstrated that the MSI1/TNS3/RhoA-GTP axis controls migration of GBM cells. MSI1 binds to the 3′UTR region of TNS3 mRNA to inhibit its translation (Fig. 4G,K), which leads to increase in cell migration. (Fig. 5B). One known effector of TNS3 is the small GTPase RhoA. Rho GTPases play important roles in regulating cytoskeletal rearrangements, cell motility, cell polarity, axon guidance, vesicle trafficking^34^. Alterations in Rho GTPase signaling contribute to malignant transformation^35^. We demonstrate that inhibition of TNS3 expression, by either overexpression of MSI1 or direct knock-down of TNS3, promotes the activation of RhoA (Fig. 5E,F) and that level of RhoA-GTP mainly correlates with migration ability and morphology of GBM cells (Fig. 5B,C). The activity of RhoA is controlled by TNS3 via DLC1 (Deleted in Liver Cancer 1), a GTPase-activating protein (GAP) which promotes hydrolysis of bound GTP to GDP for the GTPases and thus shutting off these proteins^26^. TNS3 interacts with DLC1 to promote the maintenance of the inactive form of RhoA (RhoA-GDP)^26^. We hypothesize that MSI1 overexpression, and consequently TNS3 translational inhibition, or direct knock-down of TNS3 could dissociate TNS3/DLC1 complex, unlock the inactivation of RhoA and increase the level of RhoA-GTP as observed. Interestingly, we observed that the level of RhoA activation was not only strictly dependent on TNS3 but also on the global level of MSI1 expression in the GBM cells. TNS3 overexpression does not promote RhoA activation in U251-GBM cells with low MSI1 expression compared to U251-GBM MSI1-overexpressing group (Fig. 5F,G). We hypothesize hence, it is likely that MSI1 influence RhoA activation in a TNS3-independent manner. One possibility could be that overexpression of MSI1 might directly regulate the level of DLC1 protein by either directly controlling its translation or by indirectly regulating its expression. Interestingly, DLC1 loss of expression phenocopies the changes in cell morphology^36^ that we observed for MSI1-overexpressing cells (Fig. 2C,D), but whether DLC1 mRNA is a direct target of the RNA-binding protein MSI1 needs to be further investigated.

In this study, we also identified by RIP-seq multiple targets of MSI1 that are functionally involved in actin cytoskeleton organization and cell migration (Fig. 3A,B). Even the MSI1/TNS3 axis is the major pathway that controls migration of GBM cells, we cannot exclude the possibility that MSI1 might also control additional targets somehow involved in the regulation of cell migration. Many studies reported that MSI1 is involved in the translational inhibition of its targets. For instance, MSI1 blocks the expression of Numb or p21^Cip1^, but MSI1 can also increase the expression of Robo3^37–39^. It has recently been demonstrated that MSI1 can stabilize tachykinin 1 (TAC1) through directly its binding within the exon of its target^40^. Further investigations might determine whether a distinct and specific MSI1 binding pattern is preferentially associated with translational inhibition/mRNA degradation vs mRNA stabilization, and whether these additional mRNA targets of MSI1 play an active role in the regulation of cell migration.

This study not only sheds light on how MSI1 regulates cell migration but also provided evidence to use the MSI1/TNS3 expression ratio as a predictor for the survival outcome of patients. we show that patients with tumors expressing high level of MSI1 and low level of TNS3 exhibited high risk of metastasis (Fig. 6E,F) and that patients with high MSI1/TNS3 ratio (high expression of MSI1 and low level of TNS3) exhibited low survival rate compared with patients with low MSI1/TNS3 ratio (Fig. 6H). We propose that the MSI1/TNS3 pattern (MSI1^low^TNS3^high^ vs MSI1^high^TNS3^low^) could be used to better assess the prognosis of the patients. To determine the migration and invasion potential of GBM tumors is of importance to improve the current treatment and prevent the rapid evolution of the tumor (Fig. 6I).

## Methods

### Cell culture

The human GBM cell lines, 05MG, U251 and GBM8401 were cultured in Dulbecco’s Modified Eagle’s Media (DMEM, Life Technologies Inc., Carlsbad, CA, USA) with 10% fetal bovine serum (HyClone Laboratories Inc., South Logan, UT, USA), 100 units/mL penicillin, and 100 μg/mL streptomycin (Life Technologies Inc., Carlsbad, CA, USA) under standard culture conditions (37°C, 95% humidified air and 5% CO2). 05MG and U251 derived MSI1-overexpressed stable cell lines were cultured in DMEM with 10% FBS, PS and 150 g/mL G418 (Life Technologies Inc., Carlsbad, CA, USA). Subcultures were performed with 0.25% trypsin-EDTA (Sigma-Aldrich Co. LLC., St. Louis, MI, USA). Media were refreshed every two days.

### Plasmid construction

MSI1 gene were amplified and sub-cloned from human genomic DNA. The FlagMSI1 was generated by inserting a 1038-bp fragment of full-length human MSI1 cDNA into p3XFlag-myc-CMV-26 vector (Sigma, No. E 6401). The complete cDNA sequence of TNS3 (NCBI accession no. NM_022748) was amplified and cloned into pCMV6-Entry expression vector (Cat# PS100001, OriGene Technologies, Rockville, MD, USA). To construct a reporter vector containing wild-type 3′UTR of TNS3 mRNA, and 1-kb fragment from TNS3′UTR were amplified from 05MG cells by PCR and cloned into the pMIR-REPORT luciferase vector downstream of the luciferase gene. The primers used for amplification were listed in Supplemental Table I.

### Animals and tumor cell transplantation

All animals used in this study were bred and maintained according to the Guidelines for Laboratory Animals in the Taipei Veterans General Hospital under the supervision of Department of Medical Research and Education of Taipei Veterans General Hospital. For subcutaneous mouse model, the GBM cell lines were harvested, washed, suspended in PBS and subjected to subcutaneously implantation into the dorsolateral side of the flank region of 8-week-old male BALB/c nude mice (National laboratory animal center, Taipei, Taiwan). Tumor size in the subcutaneous xenograft model was measured every two days using a caliper. The average tumor volume was calculated using the following equation: V = A*B2 *0.5 (A, long diameter; B, short diameter). For orthotropic injection, 1*10^6^ human GBM cells in 5μl PBS (2*10^5^ cells/μl) were harvested, washed and subjected to intracranial injection in 8-week-old male SCID mice (National Laboratory Animal Center, Taipei, Taiwan) using stereotaxic apparatus using the following coordinates: 3 mm posterior, 2 mm lateral right to the bregma and 2 mm deep from the dura. After 63 days, the GFP-imaging analysis was used to assess tumor volume.

### Cell migration assay

The Culture-insert (Ibidi® Cat# 81176) was stuck on the bottom surface of the 12-well plates. Cells were seeded at a density of 2.5 × 104 cells/70 μl medium in each well of insert and incubated overnight. After removing the chamber, a confluent layer of cells were washed with PBS three times and replaced with 2 ml of medium. The migratory cells in the gap were photographed at the indicated time. For transwell assay, a fluoroBlok 24-multiwell Insert System with 8-mm pore size polyethylene terephthalate membrane (Corning Inc., Corning, NY) was used. Cells were seeded in each chamber with a density of 2.5 × 104 cells in 300 μl medium. The medium was removed after 24-hour incubation, and chambers were fixed by 100% methanol at room temperature for 30 mins. The membrane was stained with 50 μg/ml propidium iodide (Sigma Aldrich Co., St. Louis, MI) for 30 mins, and counted under an inverted fluorescent microscope. The migrated cell counts were normalized to the increase of cell proliferation by an additional alamar blue assay, e.g. migrated cell counts/(24-hour cell number/initial seeded cell number) = normalized migrated cells.

### Video particle tracking microrheology

Cells were seeded on 35mm glass bottom culture dishes (16235-1SG, α -PLUS, Taoyuan, Taiwan). When cell density reached to 70% confluence, the cells were micro-injected with 20 μl fluorescent carboxylated polystyrene particles (F8801, Molecular Probes, Thermo Fisher Scientific Inc., Waltham, MA) by biolistic PDS-1000/HE particle delivery system (Bio-Rad Laboratory Inc., Hercules, CA). Cells were washed twice by PBS and replaced with fresh medium after injection and incubated over 4 hrs.

The image capture was conducted by a 37 °C and CO2-controlled chamber equipped inverted fluorescence microscope (Nikon Eclipse Ti). The two-dimensional Brownian motion of intracellular fluorescence beads was recorded for 5 sec with a frame rate of 100 FPS (Frame Per Second) and an image resolution of 130 nm/pixel via 100X oil-immersion objective (Nikon, S Fluor, N.A. = 1.3) on the microscope equipped with CMOS camera (ORCA-Flash4.0, HAMAMATSU Photonics, C11440-22C). Up to 30 individual cells and their intracellular fluorescence beads was tracked to meet statistical criteria, and the images were analyzed for two-dimensional Brownian motion of intracellular fluorescence beads by way of customized Matlab program.

The intracellular viscoelasticity included the elastic modulus G′ and the viscous modulus G′′ and inferred from MSD based on a pseudo-Stokes Einstein equation^41–43^. Each bead was calculated their track base on the [*x* (*t*) and *y* (*t*), as a function of time (*t*)], the mean squared displacement (MSD) (<Δr2(τ) > = < [*x*(*t* + τ)−*x*(*t*)]2 + [*y*(*t* + τ)−*y*(*t*)]2 > , where τ is the time lag and *t* is the elapsed time^44^. Each of the bead’s track range was in 0 < α < 1, where ‘α’ is the exponent in the expression MSD =  < Δr2(τ) >  = Aτα, then reject the beads that showed non-Brownian motion, were reserved for the calculation of G′ and G″.

### Image analysis

The cells were stained with phalloidin for the analysis of intercellular F-actin fibers orientation angles. The fluorescence images were captured by a Zeiss LSM-700 confocal microscope with a 20X objective. The F-actin orientation was analyzed by customized Matlab programs. The algorithm thresholds fluorescence images of F-actin to establish binary images including segmented F-actin fibers and quantify their orientation angles. These binary images were convolved with linearly-varying kernels in the x and y directions to gain smooth maps of the two components of the image intensity gradient, Gx and Gy and the local orientation of the fibers was obtained as ${\rm{\phi }}=\,\tan \,-1(\frac{{\rm{Gy}}}{{\rm{Gx}}})$. The segmented fibers were color-coded to represent the F-actin angular distribution. These results were quantitatively their aspect ratio (the ratio of the length of major to minor axes) show with Kurtosis coefficient (Chen, 2016, Bio- chemical and physical characterizations of mesenchymal stromal cells along the time course of directed differentiation) of the F-actin orientations, using customized Matlab programs.

### Gene expression array and Bioinformatic Analysis

For gene expressive microarray, total RNA was extracted as the previous description and the extracts were subjected to hybridize with Agilent SurePrint G3 human whole genome gene expression chip (Agilent Technologies, Santa Clara, CA, USA). Gene expression array data Genetic network construction was performed by Ingenuity Pathway Analysis (IPA) software (Qiagen, Hilden, Germany). For Ribonucleoprotein immunoprecipitation sequencing (RIP-seq), the total RNA was extracted and the cDNA library was constructed following the manufacturers instruction (TruSeq, Illumina Inc., San Diego, CA). The 50-bp single-end sequencing was performed by Illumina HiSeq. 4000 (Illumina Inc., San Diego, CA) with 20 million reads per sample. The cutoff was performed by 5log2 ratio and 2286 genes were annotated as gene list for further analysis. Genetic function and gene enrichment analysis were concluded by DAVID Bioinformatics Resources (http://david.abcc.ncifcrf.gov/). The gene ontology (GO) terms was further summarized by amigo2 (http://amigo2.geneontology.org/amigo).

### RNA-binding protein immunoprecipitation and RNA ChIP assay

Magna RIP kits (Millipore, Cat#17-700) was used for RNA-binding protein immunoprecipitation and RNA extraction following the manufacturer’s instruction. For immunoprecipitation, primary antibodies against the following proteins were used: anti-Musashi1 (Abcam, Cat#ab52865), anti-Rabbit polyclonal IgG (Millipore, Cat #12-370). RNA ChIP-IT kit (supplier, Cat#53024)^45^ were used for studying precise RNA-protein interactions. Cells were fixed by 1% formaldehyde for 5 mins. Glycine was added to the sample to make final concentration of 0.125M and incubated for 5 mins at room temperature to stop fixation. Cells were washed and pelleted by 1000 rpm centrifugation at 4 °C for 5 mins. Cells were then resuspended in ice-cold Complete Lysis Buffer, incubated on ice for 30 mins, and subjected to centrifugation at 5000 rpm 4 °C for 10 mins. Supernatant was discarded and resuspend the pellet in Complete Shearing Buffer. Samples were sonicated to shear the chromatin using Bioruptor® for 1 to 4 run of 5 cycles: [30 seconds “ON”, 30 seconds “OFF”] each (20 cycles). Samples were span at 12,000 rpm for 10 minutes. The supernatant, except the upper lipid layer, was collected. DNase I (10 μl for each sample) was added in the sample for a 20-min incubation at 37 °C, and the reaction was stopped by adding 10 μl of 0.5 M EDTA before performing the IP. For IP: First, the Dynabeads Protein-G was incubated with 2.5 μl antibody 30 mins at 4 °C. Next, 1 mg of protein lysis was incubated with protein-G-conjugated-antibody beads overnight at 4 °C. Dynabeads Protein-G was separated by Complete RNA-ChIP Elution Buffer by 15-mins rotation on the end-to-end rotor at room temperature. Transfer the supernatants and add 2 μl of 5 M NaCl and 2 μl of proteinase K to each sample to digest the proteins at 42 °C for 1 hr. Samples were incubated at 65 °C for 1.5 hrs to reverse the cross-links. RNA was extracted with phenol/chloroform/isoamyl alcohol, dissolved in 20 μl of KAPA distilled water, and used as a source of RNA for End point RT-PCR analysis (KAPA SYBR FAST Universal One-step qRT-PCR kit, KR0393). All primers used in this assay were listed in Suppl. Table III.

### Statistical analysis

Data are expressed as the mean ± SD from at least three independent experiments. The statistical analysis was performed using student’s T-test. The difference was considered significant when p ≤ 0.05 or p ≤ 0.01.

## Electronic supplementary material

Supplementary information
