# Supplementary material for: Musashi-1 Enhances Glioblastoma Cell Migration and Cytoskeletal Dynamics through Translational Inhibition of Tensin3
Source: Sci Rep. 2017 Aug 18;7:8710. doi: 10.1038/s41598-017-09504-7 (PMC5562834; doi:10.1038/s41598-017-09504-7)

Musashi-1 Enhances Glioblastoma Cell Migration and Cytoskeletal Dynamics through Translational Inhibition of Tensin3

Hsiao-Yun Chen[1]*, Liang-Ting Lin[2,3,6]*, Mong-Lien Wang[2,4], Benoit Laurent [7], Chih-Hung Hsu[7], Chih-Ming Pan[4,9], Wan-Ru Jiang[3], Pau-Yuan Chen[8], Hsin-I Ma[10], Yi-Wei Chen[1,11], Pin-I Huang[1,11], Arthur Chiou[5], Shih-Hwa Chiou[1,2,3,4,12]

Supplementary Information

SUPPLE. FIGURE LEGEND

Supple. Figure 1. MSI1 promoted GBM cells migration.

(A) 05MG cells transiently transfected with siRNA against MSI1 (siMSI1#1 and siMSI1#2) or scrambled siRNA control (siCON), and (C) U251 cells transiently transfected with Flag-tagged MSI1 (FlagMSI1) or Flag control (Flag) were subjected to Western blotting to assess MSI1 protein levels. (B) MSI1-depleted 05MG cells and (D) MSI1-overexpressed U251 cells were subjected to a 24-hour Transwell migration assay. The number of migratory cells were counted and presented as percentages relative to the controls. All values are mean + SEM. *, $P < 0.05$ (relative to the control group).

Supple. Figure 2. MSI1-regulated genes indicated the relationship with cell migration pathway through bioinformatic analysis

(A) The gene list was analyzed by DAVID (https://david.ncifcrf.gov/) and illustrated by QuickGO (https://www.ebi.ac.uk/QuickGO/) based on the significance of each GO term using False discovery rate (FDR) as the criterium. (B) Both gene expressive profiles from microarray and RIP-seq were demonstrated as log ratio and FPKM on a X-Y plot and the genes with higher correlation between the two axes were highlighted and further subjected to Gene Ontology analysis;

Supple. Figure 3. MSI1 suppressed TNS3 translation through binding to 3'UTR of TNS3.

(A) 05MG cells transiently transfected with siRNA against MSI1 (siMSI1#1 and siMSI1#2) or scrambled siRNA control (siCON) were subjected to Western blotting to assess TNS3 and MSI1 protein levels. (B) Schematic diagram of human TNS3 mRNA and the designed locations of amplified regions for RNA-Chip assay. (C) Schematic representation of the reporter constructs containing the firefly luciferase coding sequence fused to wildtype or mutated TNS3-3'UTR. The predicted binding site are noted in colored ovals and the mutated residues are marked with a cross in the ovals. (D) 05MG cells were transfected with siMSI1 for 24 hours or (E) U251 cells were transfected with FlagMSI1 for 24 hours and treated with 10 μg/ml of cycloheximide (CHX) to block protein synthesis. Cellular proteins were harvested at indicated time points after CHX treatment, and analyzed by Western blot (top). The intensity of TNS3 blot at each time point were calculating and presented as relative percentage in comparison to the 0 hours' time point (Bottom).

Supple. Figure 4. MSI1 promoted cell migration through regulation TNS3/RhoA-GTP signaling pathway in GBM.

(A) MSI1- and TNS3-depleted 05MG cells were subjected to a TransWell migration assay. (B) MSI1- and TNS3-depleted 05MG cells were subjected to wound-healing assay for 10 hours. (C) MSI1 and TNS3 protein levels were assessed in 05MG cells transiently transfected with siMSI1#1 and/or siTNS3#1 using Western blotting. Wound healing assay was performed in MSI1- and TNS3-depleted 05MG cells, and the number of migratory cells were calculated and shown in the graphs. (D-E) MSI1- and TNS3-depleted 05MG cells were seeded in 3.5-cm plates before using time lapse microscopy for single-cell tracking (1 frame/5 min for 24 hr). The tracks of single cell were shown in the left panel and the mean tracks of 15 cells were shown in the middle panel. The mean motility and speed of cells was determined by the manual tracking plugin of Image J in three independent analyses and shown as bar charts in the right. (F) MSI1- and TNS3-depleted 05MG cells were stained for F-actin (red) and DAPI (blue). The ratio of mean aspect (defined as the ratio of the length of major and minor axes, AR) were shown in the right (n = 100). (G) MSI1 regulated cell viscoelasticity through inhibiting TNS3. The elastic modulus (G') and viscous modulus (G''; at 10 Hz) were analyzed by MetLab software. Data are expressed as mean ± SEM. All values are mean + SEM. *, $P < 0.05$ (relative to the control group). #, $P < 0.05$ (relative to the siTNS3

group).

Supple. Figure 5. Uncropped blots from original scanned raw data of designated figures.

MATERIALS AND METHODS

Transfection

Plasmid transfection was carried out using jetPEI DNA transfection reagent (Polyplus jetPEI®, Cat #101-10, DNA Transfection reagent, Huntingdon, UK) according to manufacturer's instructions. siRNA against MSI1 (siMSI1#1: SASI_Hs01_00145278, siMSI1#2: SASI_Hs01_00145277), TNS3 (siTNS3#1: SASI_Hs01_00166414, siTNS3#2: SASI_Hs01_00166415) and scrambled siRNA control (MISSION® siRNA Universal Negative Control #1) were purchased from SIGMA siRNA smart pools. Transient siRNA transfection was carried out using INTERFERin siRNA transfection reagent (Polyplus INTERFERin®, Cat #409-10, siRNS transfection reagent) according to the manufacturer's instruction. Cell-based experiments were performed after 48 hours of incubation. The information of plasmids was listed in Suppl. Table 1.

Western blotting

All total protein lysates were collected with RIPA buffer (Millipore, Cat #20-188, RIPA Lysis Buffer, 10X) containing 1% protease inhibitor (SIGMA, Cat #05892791001 ROCHE, cOmplete™ ULTRA Tablets, Mini, EDTA-free, EASYpack Protease Inhibitor Cocktail). Equal amount of total proteins was separated by electrophoresis on SDS/PAGE. After the proteins were transferred onto a polyvinylidene difluoride membrane (Millipore, Cat # IPVH00010, Immobilon-P Membrane, PVDF, 0.45 µm, 26.5 cm x 3.75 m roll), the blots were incubated in blocking buffer (1 X PBST and 5% skim milk) for 1 hour at room temperature and then hybridized with primary antibodies overnight at 4°C, followed by incubation with horseradish peroxidase-conjugated secondary antibody for 1 hour at room temperature. The blots were obtained by X-ray film exposure. The primary antibodies used in this study were anti-Flag (Sigma-Aldrich Co. LLC., St. Louis, MI, USA, F1804), anti-Musashi1 (cell signaling, #5663), anti-TNS3 (SIGMA, #SAB4200416), anti-RhoA (cell signaling, #2117) and anti-β-actin (sigma, A5316). The secondary antibody used in this study were anti-Rabbit-HRP (cell signaling, #7074) and anti-Mouse-HRP (cell signaling, #7076).

Luciferase reporter assay

U251 cells were co-transfected with pMIR-control constructs, TNS3-3'UTR-WT, TNS3-3'UTR-mut1, TNS3-3'UTR-mut2 and Flag-control or Flag-MSI1 using jetPEI DNA transfection reagent in 24-well plate. The pMIR-REPORT β-gal control Plasmid, containing β-gal luciferase, was used as an internal control. The cells were cultured at 37 °C in 5 % CO_2 for additional 24 hrs. The cells were harvested and the luciferase activity was measured using dual luciferase assay system (Thermo, Cat#T1003, Dual-Light® luminescent reporter gene assay system) according to the manufacturer's instructions.

Quantitative real-time PCR (qPCR)

Total RNA were isolated from GBM cells using TRlzol (Life Technologies Inc., Carlsbad, CA, USA) followed by phenol:chloroform purification and ethanol precipitation. Reverse transcription were carried out using SuperScript III reverse transcriptase (Life Technologies Inc., Carlsbad, CA, USA). Oligonucleotides (Suppl. Table II) were designed using Primer Express 2.0 (Applied Biosystems, Foster City, CA, USA). The real-time-PCR was performed with power SYBR Green PCR Master Mix (Applied Biosystems, Foster City, CA, USA) according to manufacturer's instruction. Signals were detected with 7900HT Fast Real-time PCR system (Applied Biosystems, Foster City, CA, USA). The expression level of each gene was normalized to endogenous beta-actin and experimental control through ΔCt methods.

Immunofluorescence (IF) staining

Cells were sub-cultured on glass coverslips for 24 hrs. The cells were fixed with 4 % paraformaldehyde for 10 mins, permeabilized with 0.1% Triton X-100 for 10 mins, NP-40 for 10 mins, and incubated with blocking buffer (5% BSA) for 1 hour at room temperature. The samples were immune-stained with the indicated primary antibodies overnight at 4°C, followed by FITC-labeled or PE-labeled secondary antibodies for imaging. The primary antibodies used in this study were anti-F-Actin (Thermo, Cat # R415, Rhodamine Phalloidin), DAPI (SIGAM, Cat #D9542).

Immunohistochemistry staining and immunoblotting (IHC)

Tumor specimens from mice and GBM patients were fixed with 4% paraformaldehyde

(Sigma-Aldrich Co., St. Louis, MI, USA). Sections were deparaffinized and rehydrated, and subjected to antigen retrieval by boiling for 30 mins in 10 mmol/L (pH 6) citrate buffer (Sigma-Aldrich Co., St. Louis, MI, USA). Sections were cooled in TPBS for 10 mins before treating with 3% H_2O_2. Sample were blocked in 5 mg/ml BSA (Sigma-Aldrich Co., St. Louis, MI, USA) for 30 mins before hybridizing with 1/100 diluted primary antibodies anti-Flag (Sigma-Aldrich Co. LLC., St. Louis, MI, USA, F1804), anti-TNS3 (SIGMA, #SAB4200416) overnight at 4°C. Signals were amplified by the TSA Biotin System (PerkinElmer, Waltham, MA) following the manufacturer's instruction and the samples were counterstained with hematoxylin. The sections were examined under Olympus BX61 microscope (Olympus Corp., Tokyo, Japan), and three field of views were randomly selected and photographed for evaluation.

Suppl. Table I. List of the primers used for plasmid construction

Name	Sequence (5'-3')
MSI-F	ATGGAGACTGACGCGCCCCAGCCCG
MSI1-R	TCAGTGGTACCCATTGGTGAAGGCT
MSI1-F-HindIII	AGAAGCTTATGGAGACTGACGCGCCCCAGC
MSI1-R-BamHI	AGGATCCTCAGTGGTACCCATTGGTGAAGG
TNS3-3’UTR-F	TTCGTATCAAAGGTCATGATTGGT
TNS3-3’UTR-R	GGGTACACAAATACCGTCTCACGG
TNS3-3’UTR-MutA-F	CGTCTTCTCTGCCTTAGGCAGGCATATTTTTACTTT
TNS3-3’UTR-MutA-R	GAAAGTAAAAATATGCCTGCCTAAGGCAGAGAAGAC
TNS3-3’UTR-MutB-F	CTATAAATCTTTTGTCGCAGCATTTTCTCTGCTCC
TNS3-3’UTR-MutB-R	GGAGCAGAGAAAATGCTGCGACAAAAGATTTATAG

Suppl. Table II. Sequences of the primer used for real-time PCR analysis

Gene Name	Forward sequence	Reverse sequence
MSI1	TTGACAAAACCACCAACCGG	CCTCCTTTGGCTGAGCTTTCTT
TNS3	AGACGATGACCCCTGGCTAT	CGTGGTTCACGTCCTCCTTT
β-actin	GCGTGACATTAAGGAGAAG	GAAGGAAGGCTGGAAGAG
GAPDH	AGAAGGCTGGGGCTCATTTG	AGGGGCCATCCACAGTCTTC

Suppl. Table III. Sequences of the primer used for RNA ChIP analysis

TNS3 mRNA Seq.	Forward sequence	Reverse sequence
813-1111	TGCAGCCTTCCCAAAAACGG	CGAGCGGTATTTCTTGTGGTAG
1243-1523	ATTAGTCTTCTCTGCCACGCC	CAGACAAGGTGTGGTCACTGT
1760-2032	GCTGCTCTGAAGGATCGGG	TCGACTCCCAAAAGTCCGCT
2310-2561	CACATCCCCCTGACACACAG	TGTCTGGAGACACAGAGCCA
2733-3021	AATGTGCATGGGGAAAGGCT	CAGCTTCGTGGTTCACGTC
3329-3559	TCAGAGCTCCAGGCTCCTTT	CCACCGTGAGAAAGTTGTGG
3779-4029	CCCAAATGTCCTTCCCGACT	TGGCCAAATCTCCAGCTTTCT

4229-4526	GAAAGTTCTCCCCAGACGGC	ACTTCCTGTCTTGTGGGTCC
4858-5102	AACATCACCGTGAATTGGCCT	AAGCATGGCAGTCACTCGG
5271-5448	CGAAGGTTTGTGCAAGTCAGG	TTCTCCTCTCGGTCCTCACAG
5726-5935	TTCCCCGATTGTGGATGACT	CACAACCACACATGGTCCCC
6285-6514	CCATTCTTAGGCCACACTGGT	AATGAGGCACTTGGTTTGGTG
6778-7027	AGTGCAGGCCAGGTTCCTTA	ATTCCCCGATGCCCTTGACC
7291-7548	CCTGGGGAGTAAATGCGGAG	AAGGAGCAGAGAAAATGACTATACA

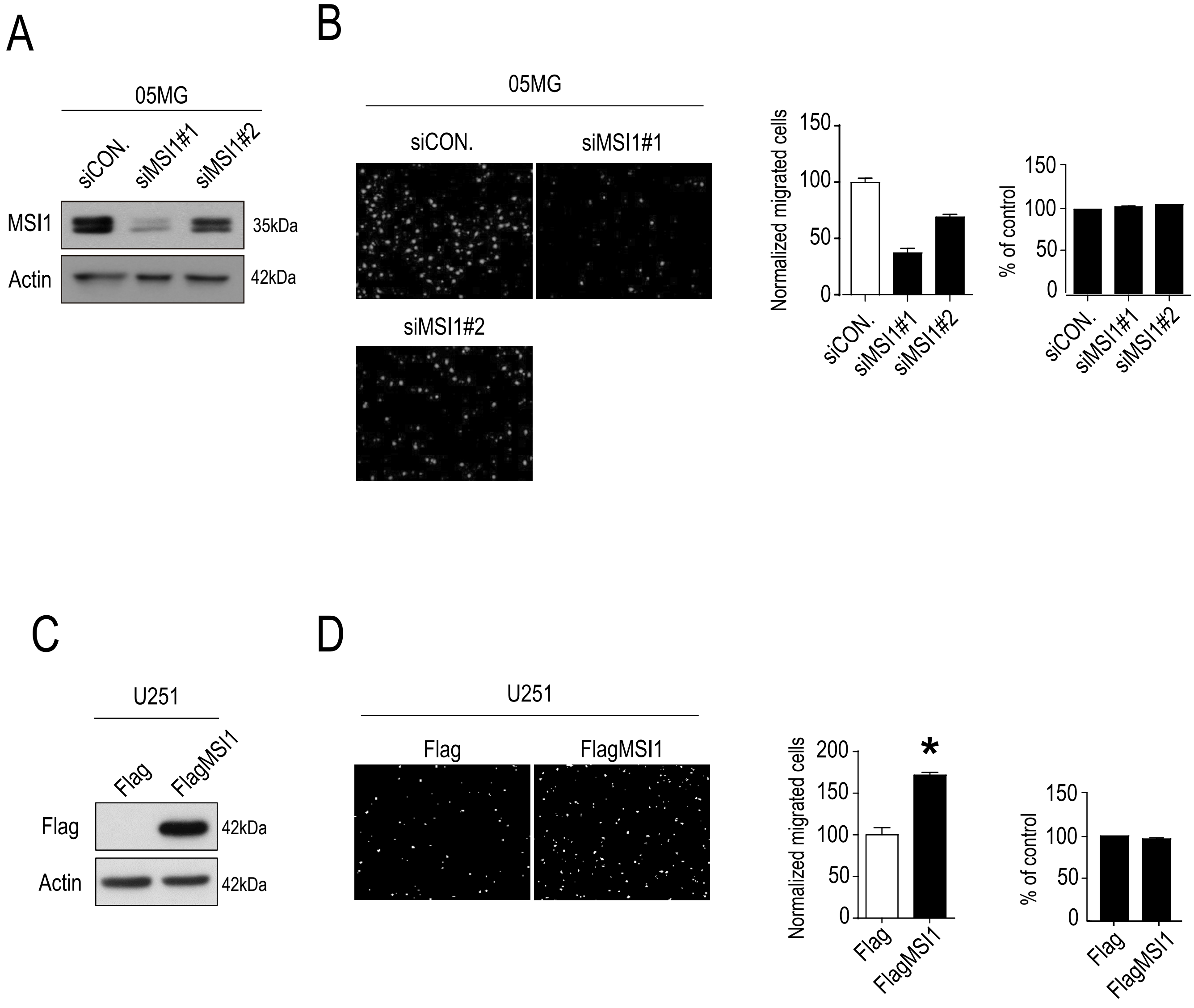
A
05MG
siCON.
siMSI1#1
siMSI1#2
MSI1
35kDa
Actin
42kDa
B
05MG
siCON.
siMSI1#1
siMSI1#2
Normalized migrated cells
150
100
50
0
% of control
C
U251
Flag
FlagMSI1
Flag
42kDa
Actin
42kDa
D
U251
Flag
FlagMSI1
Normalized migrated cells
200
150
100
50
0
*
% of control

Supple. Figure 2

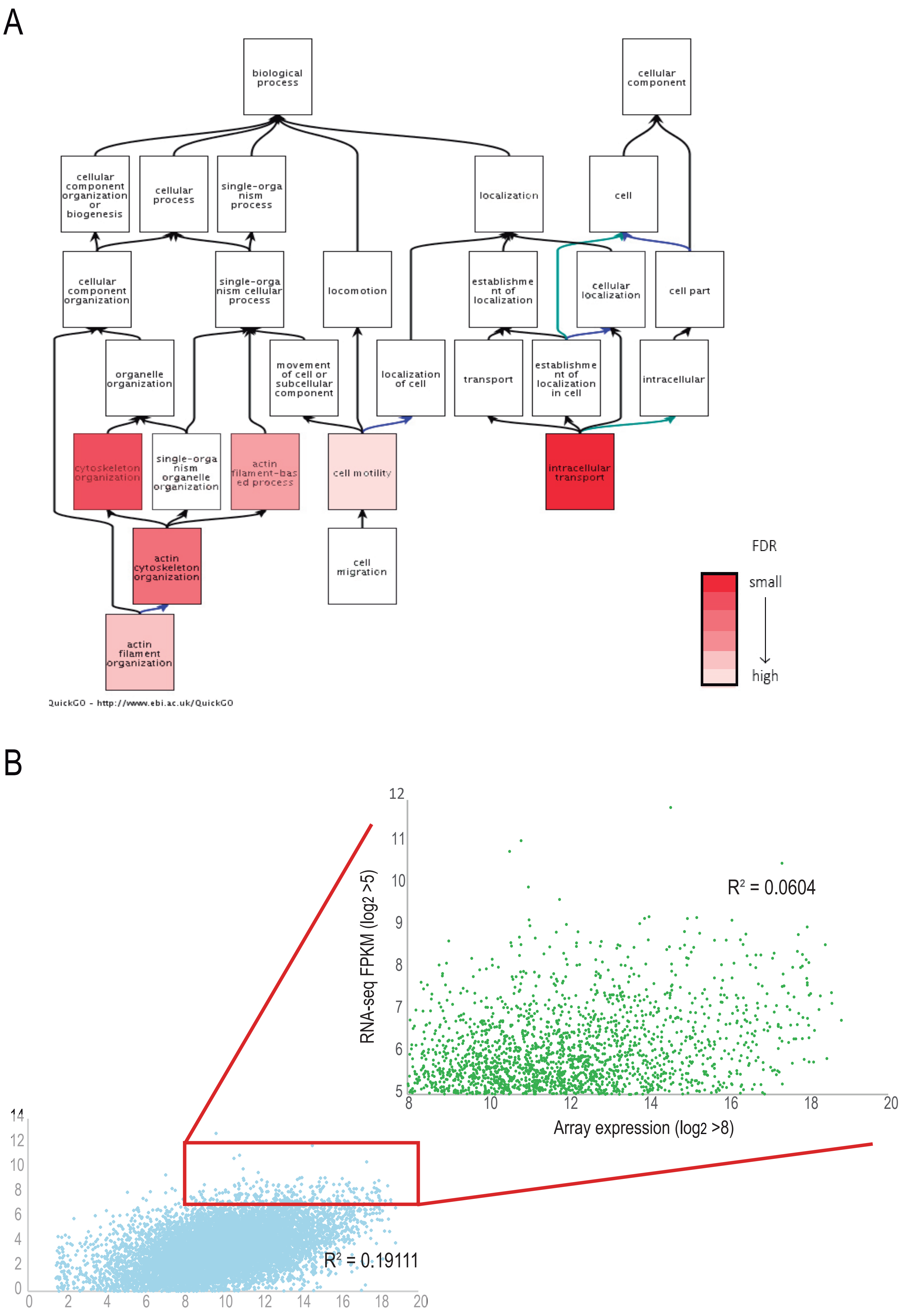

Supple. Figure 3

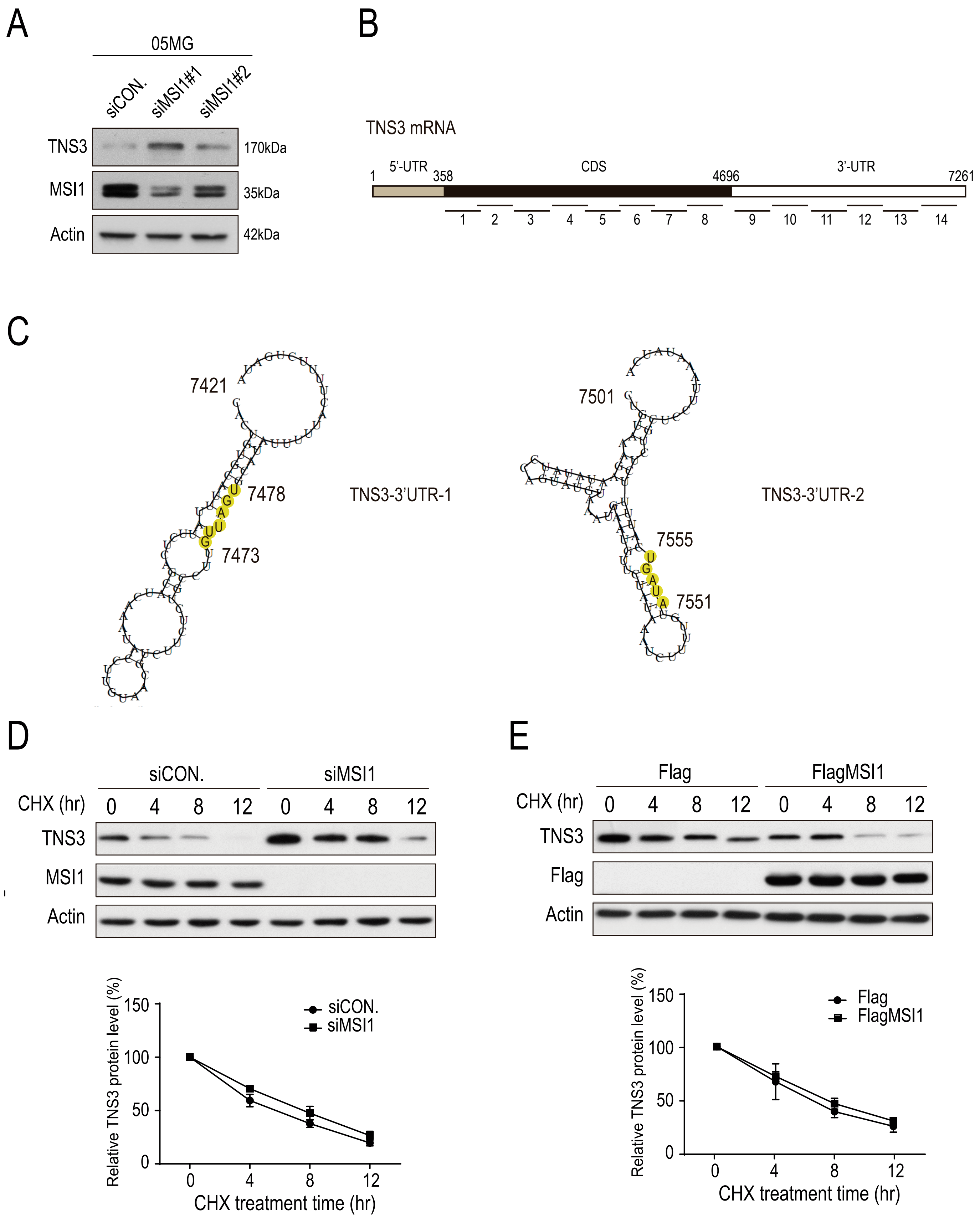

Supple. Figure 4

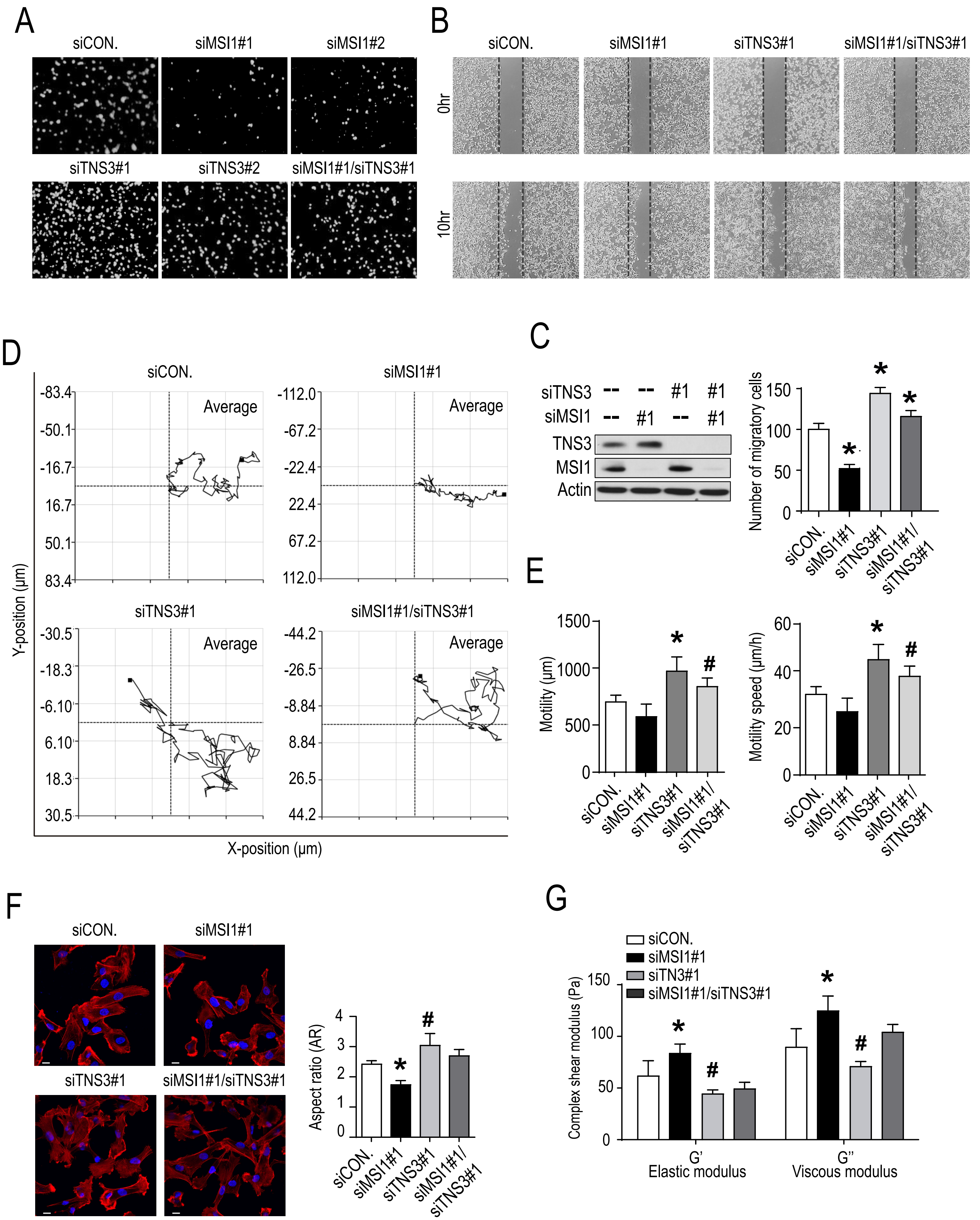

Supplement: Supplementary file 1 — Supplementary information [file 41598_2017_9504_MOESM1_ESM.pdf]
